# Household spraying in cholera outbreaks: Insights from three exploratory, mixed-methods field effectiveness evaluations

**DOI:** 10.1371/journal.pntd.0008661

**Published:** 2020-08-31

**Authors:** Karin Gallandat, Annie Huang, Justine Rayner, Gabrielle String, Daniele S. Lantagne

**Affiliations:** 1 Department of Civil and Environmental Engineering, Tufts University, Medford, Massachusetts, United States of America; 2 Department of Disease Control, London School of Hygiene and Tropical Medicine, United Kingdom; Wayne State University, UNITED STATES

## Abstract

Household spraying is a commonly implemented, yet an under-researched, cholera response intervention where a response team sprays surfaces in cholera patients’ houses with chlorine. We conducted mixed-methods evaluations of three household spraying programs in the Democratic Republic of Congo and Haiti, including 18 key informant interviews, 14 household surveys and observations, and 418 surface samples collected before spraying, 30 minutes and 24 hours after spraying. The surfaces consistently most contaminated with *Vibrio cholerae* were food preparation areas, near the patient’s bed and the latrine. Effectiveness varied between programs, with statistically significant reductions in *V*. *cholerae* concentrations 30 minutes after spraying in two programs. Surface contamination after 24 hours was variable between households and programs. Program challenges included difficulty locating households, transportation and funding limitations, and reaching households quickly after case presentation (disinfection occurred 2–6 days after reported cholera onset). Program advantages included the concurrent deployment of hygiene promotion activities. Further research is indicated on perception, recontamination, cost-effectiveness, viable but nonculturable *V*. *cholerae*, and epidemiological coverage. We recommend that, if spraying is implemented, spraying agents should: disinfect surfaces systematically until wet using 0.2/2.0% chlorine solution, including kitchen spaces, patients’ beds, and latrines; arrive at households quickly; and, concurrently deploy hygiene promotion activities.

## Author summary

Cholera remains a global health concern, with an estimated 2.9 million cases and 95,000 deaths per year. Household spraying is an outbreak response activity where the houses of cholera patients are disinfected by spraying chlorine to interrupt cholera transmission within households. The effectiveness and appropriateness of this intervention have been questioned but it remains widely implemented. We conducted three mixed-methods evaluations of household spraying programs in the Democratic Republic of the Congo and in Haiti. By sampling surfaces before, 30 minutes and 24 hours after spraying, we were able to identify household surfaces that were consistently the most contaminated at baseline (namely food preparation areas, patients’ beds, and latrines). We also found that spraying chlorine could inactivate bacteria on household surfaces but that effectiveness was inconsistent, likely due to differences in spraying protocols between programs such as the amount of chlorine applied onto surfaces. Through key informant interviews, structured observations and household surveys, we also gained insights into the advantages and challenges of each program. This work contributes to the scientific evidence base necessary to optimize cholera response strategies; the combination of microbiological and qualitative information allowed us to formulate concrete recommendations for outbreak response programs.

## Introduction

Infection with toxigenic *Vibrio cholerae* O1/O139 bacteria can cause profuse watery stool and vomiting and, if untreated, can result in severe dehydration and death within hours [1,2]. Cholera outbreaks primarily occur in regions lacking access to adequate water, sanitation and hygiene (WASH) services [3]. Population displacements and conflicts enhance vulnerability to cholera [4–9]. In 2018, 34 countries reported cholera [10] and the global cholera burden is estimated to 2.9 million cases and 95,000 deaths per year [11]. The Global Task Force on Cholera Control (GTFCC) aims to reduce cholera deaths by 90% by 2030, with timely outbreak detection and response as the first step towards cholera elimination [12].

Cholera has traditionally been considered as waterborne; however, there is a growing body of evidence suggesting that person-to-person transmission within households (via contaminated food, objects, or direct contact) is important [13,14]. For example, individuals living within 50 meters of a cholera case are 23–56 times as likely to contract cholera as those further away [15]; person-to-person transmission has been estimated to account for 41–95% of transmission in modelling studies [15–18] and, mean infection risks of 3.7–8.2% were associated with fecal shedding of *V*. *cholerae* among household contacts of cases, compared to infection risks of 2.0–3.4% from community water sources over 11 days [19]. Among the potential cholera transmission pathways, it is thus plausible that contaminated surfaces or objects (“fomites”) contribute to transmission, particularly for household contacts of cholera cases.

The likelihood of disease transmission via fomites depends on the: 1) number of pathogens shed by infected individuals; 2) number of pathogens required to cause disease (infectious dose); 3) persistence of pathogens on surfaces; and, 4) resistance of pathogens to disinfection [20]. For cholera specifically, stool from infected individuals can contain up to 10^9^ *V*. *cholerae/*mL; stool from asymptomatic individuals (who may represent up to 80% of infected individuals) can contain up to 10^3^ *V*. *cholerae*/mL [1,2]. The infectious dose for cholera is typically estimated between 10^5^ and 10^8^ bacteria [21], however transmission risk via fomites may be enhanced during the hyperinfectious *V*. *cholerae* stage (observed for at least 5 hours after shedding by an infected individual), where the infectious dose could be <100 bacteria [22].

To our knowledge, *V*. *cholerae* persistence on household surfaces has not been evaluated. In laboratory tests, *V*. *cholerae* bacteria lose culturability on surfaces within 1.5–4 hours but can persist for a week on dry surfaces in a “viable but nonculturable” (VBNC) stage [23]. VBNC cells cannot be detected using culture-based methods, however they may remain infectious [24,25]. Lastly, regarding surface disinfection efficacy, only one relevant laboratory study was identified, where >6 log inactivation in *V*. *cholerae* on glass and aluminum surfaces was measured after spraying 0.6% (6,000 mg/L) sodium hypochlorite with a 15-minute exposure time; and, on wood surfaces, two disinfectant applications and 30 minutes were required to achieve the same efficacy [26].

In cholera outbreaks, household spraying is a commonly implemented intervention where cholera patients’ homes are disinfected with chlorine, with the aim to reduce the immediate risk of cholera transmission to other household members via surfaces and/or objects contaminated with *V*. *cholerae*. To our knowledge, the first record of household spraying was in Taiwan in 1962 [27]. Recently, household spraying has been questioned and in a recent review of international cholera guidelines, it was identified as one of few interventions explicitly not recommended by four agencies [28]. Concerns related to household spraying included [29,30]: 1) lack of evidence of effectiveness of a one-off chlorine spraying process at reducing *V*. *cholerae*, as chlorine can be rapidly inactivated by organic matter present on household surfaces; 2) lack of evidence on epidemiological impact; 3) logistical constraints, as infection could spread before teams are able to disinfect; 4) that asymptomatic individuals are not identified and thus not targeted; 5) risk of stigmatization of the patient and family; 6) potential damage to household belongings by chlorine; and, 7) the amount of time and resources required to implement programs. Despite these concerns, and that no evidence was found supporting the effectiveness of household spraying in a systematic review of WASH interventions in response to outbreaks [31], household spraying is still recommended in several national guidelines and remains widely implemented in outbreak response [32–37], sometimes included as part of case-area targeted response interventions (CATI) [38,39].

To inform the ongoing discussion on household spraying, we evaluated household spraying programs in cholera outbreaks, specifically seeking to: 1) determine where *V*. *cholerae* is found on surfaces in cholera patient houses; 2) assess the immediate effectiveness of household spraying at inactivating *V*. *cholerae* on surfaces; 3) observe surface recontamination patterns within 24 hours following the intervention; and, 4) document program advantages and challenges.

## Methods

A mixed-methods protocol was developed, which included key informant interviews, structured observation of spraying with chlorine measurement, household surveys, and surface sampling. All tools including key interview and observation guides, and the survey questionnaire are provided in S1 File.

### Ethics statement

This protocol was approved by the Tufts Social, Behavioral, and Educational Research (SBER) Institutional Review Board (#1712002). In Haiti, approval was granted by the National Bioethics Committee (#1819–7). In the Democratic Republic of the Congo (DRC), in the absence of an SBER process, ethical and cultural appropriateness were confirmed by a certified, independent medical researcher. Written consent was obtained from all participants prior to starting study activities.

### Site selection

Solicitations for program participation in the research were sent via the Global WASH Cluster and Cholera Platform e-lists, followed by regular communication with humanitarian WASH partners, including personal contacts. Potential evaluation sites had ongoing outbreaks with spraying expected to continue for 4 weeks. After a spraying program was identified for potential inclusion, approvals were obtained from: implementation partners, local and Tufts ethics boards, and Tufts Global Operations before Tufts staff deployment to complete the evaluations.

### Key informant interviews

Key informant interviews (KII) were conducted in the local language with program coordinators and spraying agents. Semi-structured interview guides consisted of 35 and 49 questions for coordinators and spraying agents, respectively. Coordinators were asked questions about their experience in cholera outbreaks; program organization and logistics; household spraying protocols; staff selection and training; and, perception of spraying interventions. Program staff interviews addressed participants’ professional background; cholera prevention knowledge; training; household spraying protocol; daily activities, opportunities and challenges; and, reception of the intervention by beneficiaries. Interviews were audio-recorded with participant consent, and transcribed in Microsoft Word to extract information.

### Structured observations with chlorine measurement

Beginning at the spraying team base, study team members accompanied spraying agents to houses. Activities, including chlorine solution preparation and spraying, were observed at each house; structured guides were used to record observations. Chlorine solution pH and concentration were measured immediately before spraying using a pH-meter (Apera EC60, Switzerland) and an iodometric titration test kit (Hach method #8209, USA), respectively. For ethical reasons, if solutions were <70% of the expected concentration, the study team requested chlorine be added to obtain the target concentration. As such, sampling results reflect the effectiveness of spraying with a chlorine solution that is within 10% of the target concentration.

### Household surveys

Participants were enrolled if an adult was present when the spraying team arrived and consented to participate. A household survey questionnaire with 75 questions addressing demographic information, hygiene habits, and cholera information was administered in the appropriate local language by a trained enumerator. Responses were recorded on paper forms, and data entered into Microsoft Excel 2016 (Redmond, WA, USA) for analysis.

### Surface sampling

At each house, a sampling floor plan was drawn and 10 surfaces representative of daily activities (e.g. kitchen floor, furniture, latrine) and materials (e.g. plastic, wood, dirt) were identified. Surface selection was standardized (to the extent possible) across houses. Samples were collected from the 10 selected surfaces at three time points (for a total 30 samples/house): immediately before spraying; 30 minutes after spraying was completed; and, 20–24 hours after spraying. A 10 cm × 10 cm stencil was used to delineate the sampling area and placed on an adjacent area of the same surface at each sampling point. Samples were collected using Sanicult swabs (Starplex Scientific, ON, Canada) in 5 mL of buffer solution, kept on ice, and transported to the field laboratory for analyses to be performed within 8 hours of collection.

### Microbiological analysis

Surface samples were analyzed for *Escherichia coli (E*. *coli)* and total coliforms, and *V*. *cholerae*. For simultaneous detection of *E*. *coli* and total coliforms, one milliliter of sample (Sanicult swab buffer) was added to a PetriFilm (3M, MN, USA) and incubated at 35°C for 24 hours. The theoretical detection limit was 5 CFU/100 cm^2^. For *V*. *cholerae* detection, 250 μL of sample was spread onto thiosulfate-citrate-bile salts-sucrose (TCBS) agar (BD, NJ, USA) and incubated at 35°C for 24 hours. Round, smooth, yellow colonies were recorded as presumptive toxigenic *V*. *cholerae* O1/O139 (hereafter termed *V*. *cholerae*); due to field testing limitations, the identification of *V*. *cholerae* was not confirmed by other means, as further detailed in Discussion. The theoretical detection limit was 20 CFU/100 cm^2^. Field laboratory results were recorded in a notebook and entered into Excel for concentration calculation and graphical analysis. MATLAB R2017a (MathWorks, MA, USA) was used for statistical tests. Friedman’s test was used to compare bacterial counts obtained before, 30 minutes, and 24 hours after spraying. If statistically significant differences between time points were detected (p<0.05), Wilcoxon’s signed rank test was performed with Bonferroni correction for multiple comparisons (p<0.017 considered significant).

## Results

Household spraying programs were evaluated in June and July 2018 in two locations in DRC, and in October 2018 in Haiti. In total, 18 key informant interviews were conducted, 14 households were enrolled, and 418 surface samples were collected (Table 1).

**Table 1:** Key characteristics of spraying programs evaluated.

	Program A	Program B	Program C
**Evaluation date**	June 2018	July 2018	October 2018
**Environment**	Urban	Urban / semi-urban	Urban
**Cholera context**	Endemic	Epidemic	Endemic
**Start of spraying activities**	2008	April 2018	2014
**Spraying agents**	3 (+6 “back-up”)	9	11
**Supervision**	Local health authorities	NGO	NGO
**Spraying agents’ base**	Diarrheal disease treatment center at the general hospital	Cholera treatment centers, units, and oral rehydration points	NGO office
**Mode of transport**	On foot or motorcycle	Motorcycle (with driver)	4x4 vehicle (with driver)
**Coverage objectives**	Patient house + 5 latrines around	Patient house + 20 neighbors	Patient house + 20–30 neighbors
**Other activities**	Basic hygiene promotion to case household and neighbors by spraying agents	Hygiene promotion & kit distribution to 5 neighbors by spraying agents or supervisor with support from 4x4 vehicle	Hygiene promotion (neighbors included), water treatment products and/or antibiotic prophylaxis (case household), and 2 follow-up visits by other agents
**Chlorine type**	Calcium hypochlorite	Calcium hypochlorite	Calcium hypochlorite
**Chlorine concentrations**	0.2% for general surfaces; 2% for latrines/soiled areas	0.2% for general surfaces; 2% for latrines/soiled areas	0.2% for general surfaces; 2% for latrines/soiled areas
**Solution preparation**	At house	At base	At base

### Key informant interviews & structured observations

Information collected during interviews and observation was consolidated to describe program structure, agent training, protocols for chlorine solution preparation and spraying, and challenges, as described below.

### Program structure

Program A was implemented in an urban endemic cholera setting. Household spraying had been initiated in 2008 by the local public health authorities. The ongoing outbreak was declared in June 2016 (two years before the evaluation). The spraying team was based at the diarrheal disease treatment center and reached households located within 15 km of their base, using motorcycles or walking. The team was composed of 12 people: 3 agents for household spraying, 2 agents for healthcare facility disinfection, 1 administrative assistant, and 6 back-up agents who were not active during the evaluation. Household spraying activities were supervised by local public health authorities and funded over time by different non-governmental organizations (NGOs) participating in cholera response, in coordination with the WASH Cluster.

Program B was implemented in a large urban/semi-urban epidemic cholera context. The outbreak began in February 2018, the first in >10 years. Household spraying was initiated in April 2018, interrupted in June, and resumed in July, following a resurgence of cases, with a 45-day funding window. Nine program agents were split into four teams. Teams were based at cholera treatment centers (2), a cholera treatment unit, and an oral rehydration point. All spraying agents were paired with a motorcycle and driver.

Program C was located in a large city where spraying activities began in 2014. The eleven spraying teams (each composed of a spraying agent and a hygiene promotion agent, sometimes accompanied by a Ministry of Health representative to distribute antibiotic prophylaxis to household members of suspected cholera cases) were based at an NGO office as part of a Rapid Response Team program. Each team was paired with a vehicle and driver. Six sentinel agents were stationed at cholera and diarrhea treatment centers and reported new cases to spraying teams via WhatsApp.

Spraying teams had similar schedules in all programs. Each day began with the identification of new patients and documenting their addresses, either via healthcare staff (Programs A, B) or the sentinel system (Program C). Spraying agents then gathered their protective clothing (goggles, boots, gloves), calcium hypochlorite, spoon, and spraying equipment. Spraying solutions were prepared at the house (Program A) or at the base (Programs B and C). Programs differed in their coverage objectives. In Programs A and B, households of suspected cholera cases received the intervention, but cases were not confirmed before deployment of the spraying teams; in Program C, all households of patients with acute watery diarrhea were targeted with household spraying. Furthermore, in Program A, the cholera patient’s house and five neighboring latrines were sprayed; in Programs B and C the patient’s house and latrine, and 20–30 neighboring houses and latrines, were sprayed.

Upon arrival at a house, agents in all programs delivered a hygiene promotion session to the cholera patient’s household members and available neighbors. In Program B, hygiene kits (a safe water storage bucket, chlorine tablets, soap bars, and oral rehydration kits) were distributed to five neighboring households. Program C’s protocol included two follow-up visits, 1 and 2 weeks after spraying, to reinforce hygiene promotion messages and measure free chlorine residual concentrations in stored household water. These follow-up visits were not observed during the timeframe of the evaluation.

### Spraying agents’ training and cholera knowledge

In Program A, agents reported working as spraying agents for 3–9 years and receiving spraying training several years before the evaluation. When asked about ways to prevent cholera, all interviewed agents (n = 4) mentioned handwashing; two also mentioned water treatment and using latrines.

In Program B, only one respondent had had prior experience as a spraying agent. Spraying agents reported attending a half-day training provided by the NGO running the spraying program. Five of six respondents mentioned at least three methods of preventing cholera, including handwashing, water treatment, latrine use, and cooking/heating food.

In Program C, interviewed agents all had university degrees and had experience in the humanitarian sector before joining the spraying program. They received a 1-day training provided by the NGO running the spraying program. All interviewees mentioned at least three cholera prevention methods, including handwashing, water treatment, and latrine use.

### Chlorine solutions

In all programs, high-test calcium hypochlorite powder (HTH) was used to make 0.2% solution (for household surfaces) and 2.0% solution (for latrines and soiled surfaces [e.g. where the patient vomited]). Across programs, spoons were used to dose chlorine and a wooden stick or spraying nozzle was used to mix solutions. No program had a written protocol available for chlorine solution preparation. In Program A, agents asked for “clean” water from a household member, measured the required volume using their graduated 12-L spraying equipment, and mixed the solution on site for immediate use. In Program B, agents prepared the HTH solution at the cholera treatment center (CTC) by estimating the required volume of tap water using a container of known capacity such as a 20-L bucket; the 0.2% solution was transported in the sprayer tank (5 L) and used within 24 hours, and the 2.0% solution was kept in a 2-L opaque jerrycan, transferred into the spraying equipment as needed, and kept for up to 5–7 days. In Program C, solutions were prepared at the NGO office with tap water measured in the graduated sprayer tank (5 L); solutions were stored and transported in the equipment for use within 24 hours.

**Fig 1:**
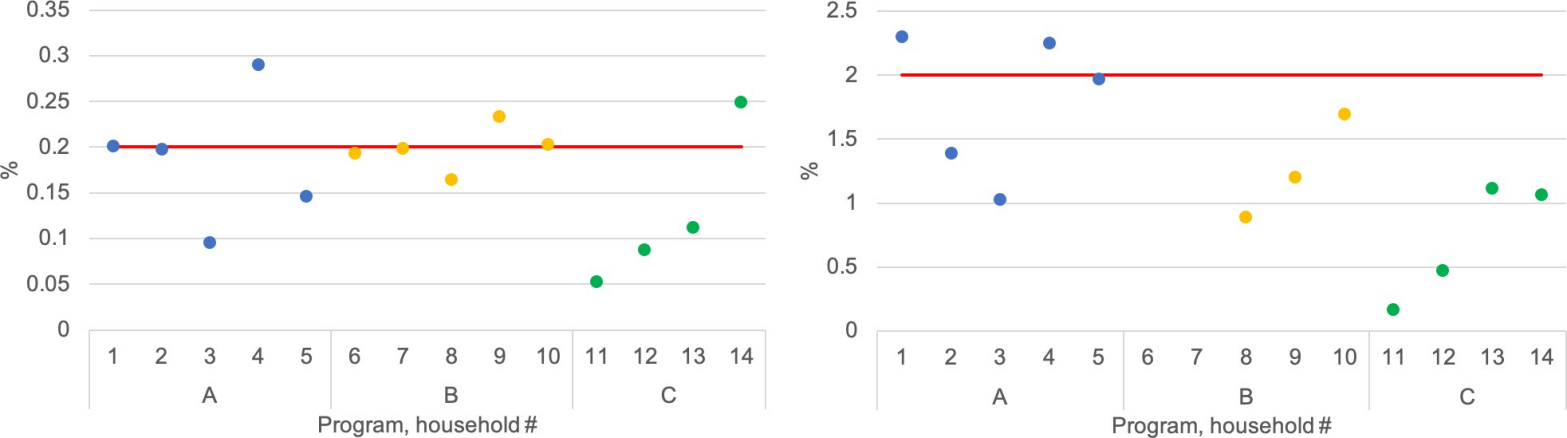
Measured chlorine concentrations (%) in spraying solutions by Program and household (#1 to #14); red line denotes target concentrations of 0.2% (left) and 2.0% (right).

Measured chlorine concentration in Program A ranged from 0.10–0.29% and 1.03–2.30% for the targeted 0.2% and 2.0% solutions, respectively; 0.16–0.23% and 0.89–1.69% in Program B; and, 0.05–0.25% and 0.16–1.10% in Program C (Fig 1). Please note, before spraying chlorine was added to meet the lowest target concentration (0.2%) in households #3, 11, 12, and 13, as per protocol. pH ranged from 8.3–8.7 and 11.4–11.7 for the 0.2% and 2.0% solutions, respectively, in Program A; 7.9–11.4 and 11.8–12.1 in Program B; and, 11.1–12.0 and 12.4–12.6 in Program C.

### Spraying protocols

Spraying agents in Program A sprayed surfaces in a systematic manner for disinfection, starting where the cholera case slept, proceeding through each room in the same direction, and spraying until surfaces were visibly wet. Spraying was completed in 5–10 minutes, using approximately 5–10 L of 0.2% chlorine solution per patient house; additional time was taken to spray five neighboring latrines using approximately 5 L of 2.0% solution in total.

Spraying agents in programs B and C did not appear to target specific areas or follow a defined protocol for household spraying. In Program B, 2–5 minutes was spent at each patient’s house using an average of 4 L total of 0.2% chlorine solution to spray 21 houses (~0.2 L per house) and 0.5–2 L of 2.0% chlorine solution was used to spray the latrines (~0.02–0.1 L per latrine). In Program C, 2–5 L of 0.2% chlorine solution were sprayed in each patient’s house over approximately 5 minutes; the volume of 2.0% chlorine solution used was not recorded.

### Identified challenges

Three concerns were raised during interviews: identification of target households, transportation, and rejection and security issues.

During the six days the study team followed Program A spraying agents, three houses listed for disinfection could not be located. Three spraying agents from Program A (50%) stated they felt patients sometimes provide incorrect addresses due to fear of stigmatization. Additionally, the study team found household identification was particularly challenging in camp settings, due to population movements and high density. During the Program B evaluation, all houses were found by spraying teams, sometimes with the help of a patient’s relative. Three interviewees (43%) from Program B still reported locating houses was difficult and time-consuming, mentioning the lack of precise addresses as an issue. They suggested the use of cell phones as a means to contact their base for guidance on locating houses.

Transportation, in particular, availability of motorcycles and fuel, was mentioned as a challenge in 5 (83%) and 3 (43%) interviews with spraying agents from Programs A and B, respectively. These concerns were linked to funding (or funding continuity) issues, explicitly mentioned by 4 interviewees (67%) in Program A and 2 interviewees (29%) in Program B.

In Program C, logistics appeared to function smoothly for the 11 cholera response teams. Concerns raised by spraying agents during interviews included security in some neighborhoods and, in three instances (60%), refusal of household spraying. The latter was attributed to fear of stigmatization or lack of trust in response team motives.

### Household surveys

The number of household members ranged from 2–13, with a mean of 6, 8.5, and 5 in Programs A, B, and C, respectively (Table 2). In Programs A and B, dirt walls (90%) and floors (100%) with metal roofs (100%) were the most common building materials; concrete walls (75%) and floors (100%) were common in Program C. Two enrolled households (20%) had electricity in Programs A and B, compared to 3 out of 4 households (75%) in Program C. Shared latrines were the most commonly available sanitation facility across programs (57%). All households 100% had soap and water for handwashing in Program A compared to half 50% in Program C; in Program B, only soap was observed at one house (20%). Floor cleaning frequencies were variable in Program A, from several times a day (40%) to daily (20%) or less (20%), while daily cleaning was most commonly reported in Programs B (80%) and C (75%). In Program A, laundry was washed in a nearby river or lake (80%), which was not possible at the other study sites. Reported laundry frequencies were variable, with 50–60% of all respondents laundering at least every 2–3 days.

**Table 2:** Selected household survey results.

	Program A n = 5		Program B n = 5		Program C n = 4	
**Demographics & context**						
N(%) Female respondent	5	(100%)	4	(80%)	4	(100%)
N(%) Respondent went to school	3	(60%)	5	(100%)	4	(100%)
Mean number of household members (range)	6	(2–13)	8.5	(7–10)	5	(3–8)
Mean number of beds (range)	1.8	(0–4)	0.2	(0–1)	2.2	(1–5)
N(%) Walls						
- Dirt	4	(80%)	5	(100%)	0	(0%)
- Concrete	0	(0%)	0	(0%)	3	(75%)
N(%) Floor						
- Dirt	5	(100%)	5	(100%)	0	(0%)
- Concrete	0	(0%)	0	(0%)	4	(100%)
N(%) Metal roof	5	(100%)	5	(100%)	3	(75%)
N(%) Electricity	1	(20%)	1	(20%)	3	(75%)
**Sanitation and reported hygiene practices**						
N(%) Latrines						
- Private	1	(20%)	0	(0%)	2	(50%)
- Shared	2	(40%)	4	(80%)	2	(50%)
- None	2	(40%)	1	(20%)	0	(0%)
N(%) Soap available for handwashing	5	(100%)	1	(20%)	2	(50%)
N(%) Water available for handwashing	5	(100%)	0	(0%)	3	(75%)
N(%) Dishwashing frequency						
- After each meal	3	(60%)	0	(0%)	3	(75%)
- Daily	1	(20%)	4	(80%)	0	(0%)
- Every 2–3 days	1	(20%)	1	(20%)	1	(25%)
N(%) Drying dishes						
- Outside	2	(40%)	4	(80%)	1	(25%)
- Inside	2	(40%)	1	(20%)	1	(25%)
- Not done	1	(20%)	0	(0%)	2	(50%)
N(%) Floor cleaning frequency						
- Several times a day	2	(40%)	1	(20%)	0	(0%)
- Daily	1	(20%)	4	(80%)	3	(75%)
- Every 2–3 days	1	(20%)	0	(0%)	0	(0%)
- No cleaning	0	(0%)	0	(0%)	1	(25%)
N(%) Laundry at river or lake	4	(80%)	0	(0%)	0	(0%)
N(%) Laundry frequency						
- Daily	2	(40%)	0	(0%)	0	(0%)
- Every 2–3 days	1	(20%)	3	(60%)	2	(50%)
- Weekly	1	(20%)	1	(20%)	2	(50%)
- Every other week or less	1	(20%)	1	(20%)	0	(0%)
**Cholera**						
Mean (range) distance from CTC [minutes]	105	(60–180)	34.5	(10–90)	- (a)	
Mean (range) time since last reported onset of cholera symptoms [days]	3.4	(2–5)	3.2	(2–4)	5.5	(5–6, n = 2)
N(%) First time having household spraying	4	(100%)	4	(80%)	3	(75%)
N(%) Appreciated about household spraying						
- Feeling safe	3	(60%)	0	(0%)	0	(0%)
- Clean house	2	(40%)	5	(100%)	3	(75%)
- Air purification	1	(20%)	0	(0%)	1	(25%)
N(%) Did not appreciate about household spraying						
- "Everything was good"	5	(100%)	5	(100%)	0	(0%)
- Doesn’t know	0	(0%)	0	(0%)	4	(100%)

(a) None of 4 respondents knew where the cholera treatment facility was located.

Survey respondents estimated they were 1–3 and 0.2–1.5 hours away from the closest CTC in Programs A and B, respectively. In Program C, no respondent knew the closest CTC. No household deaths due to cholera were reported by study participants. Overall, 2–6 days had elapsed between reported cholera/diarrhea symptom onset and spraying team arrival. Having a clean house was the most common reason why survey respondents reported appreciating household spraying (71%), followed by feeling safe (21%). When asked about what they did not like, all respondents reported “everything was good” (Programs A and B) or they did not know (Program C). Please note that, due to programmatic response protocol, two of four patients in Program C were acute watery diarrhea cases and not suspected cholera cases.

### Surface sampling: Vibrio cholerae

**Fig 2:**
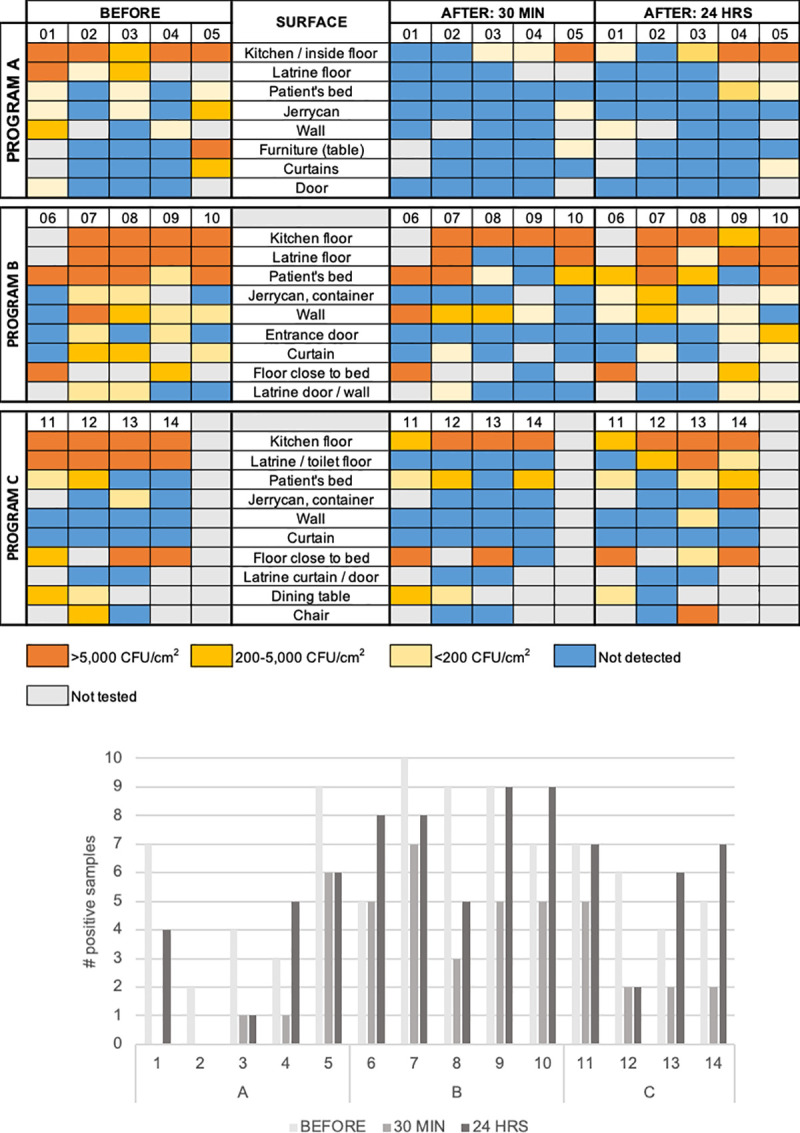
(a) *V*. *cholerae* concentrations on selected surfaces before, 30 minutes and 24 hours after household spraying. (b) Number of surfaces where *V*. *cholerae* were detected (>25 CFU/100 cm^2^), by program and household.

Before spraying, the highest *V*. *cholerae* concentrations were consistently found around food preparation spaces/floors, latrine floors, and near the patients’ sleeping areas (Fig 2A).

Overall, the number of surfaces that tested positive for *V*. *cholerae* decreased between before and 30 minutes after spraying in 13 out of 14 (93%) households. Between 30 minutes and 24 hours after spraying, an increase in the number of surfaces contaminated with *V*. *cholerae* was seen in 10 out of 14 (71%) households (Fig 2B). Results from each program are detailed below.

In Program A, before spraying, 25 surfaces across 5 houses were positive for *V*. *cholerae* (range 2–9 surfaces/household, Fig 2B). Thirty minutes after spraying, eight surfaces in three houses (range 1–6) were positive; 24 hours after spraying, 16 surfaces in four houses (range 1–6) were positive. Overall, nine surfaces had high contamination (>5,000 CFU/cm^2^) before spraying, compared to four and five surfaces 30 minutes and 24 hours after spraying, respectively. Statistically, there was decreased contamination 30 minutes (p<0.001) and 24 hours (p = 0.007) after spraying as compared to before spraying, and no statistical difference between 30 minutes and 24 hours (p = 0.064).

In Program B, 40 surfaces (5 households, range 5–10 surfaces/house) were positive for *V*. *cholerae* before spraying, compared to 25 surfaces (range 3–7) and 39 surfaces (range 5–9) 30 minutes and 24 hours after spraying, respectively (Fig 2B). Overall, 16 surfaces had high contamination (>5,000 CFU/cm^2^) before spraying, compared to 10 and 13 surfaces 30 minutes and 24 hours after spraying, respectively. Statistically, there was decreased contamination 30 minutes after spraying (p = 0.014), but not 24 hours after spraying (p = 0.804), as compared to before spraying, and a statistical difference was seen in bacterial counts between 30 minutes and 24 hours (p = 0.008). Specifically, between 30 minutes and 24 hours after spraying, observed contamination levels increased on 25 surfaces, including water containers (3), latrine floors (2), latrine walls (2), and patient’s bed (2). A decrease in contamination levels was noted on 6 surfaces, including inside walls (2).

In Program C, 22 surfaces (4 households, range 4–7 surfaces/house) were positive for *V*. *cholerae* before spraying, compared to 11 surfaces (range 2–5) and 33 surfaces (range 2–7) 30 minutes and 24 hours after spraying, respectively (Fig 2B). Overall, 13 surfaces had high contamination (>5,000 CFU/cm^2^) before spraying, compared to 5 and 10 surfaces 30 minutes and 24 hours after spraying, respectively. Statistically, no difference in bacterial counts was seen between sampling points before, 30 minutes, and 24 hours after spraying (p = 0.062).

### Surface sampling: Escherichia coli

Across programs, the highest *E*. *coli* concentrations before spraying were detected on kitchen and latrine floors, and near the patients’ sleeping areas (S1 Fig).

Overall, the number of surfaces testing positive for *E*. *coli* decreased between before and 30 minutes after spraying in 10 out of 14 (71%) households. Between 30 minutes and 24 hours after spraying, an increase in the number of surfaces contaminated with *E*. *coli* was also seen in 10 out of 14 (71%) households (S1 Fig). Results from each program are detailed below.

In Program A, before spraying, 20 surfaces (5 households, range 3–5 surfaces/house) were positive for *E*. *coli*, compared to 8 surfaces (range 0–3) and 18 surfaces (range 2–5), 30 minutes and 24 hours after spraying, respectively (S1 Fig). Statistically, there was decreased contamination 30 minutes after spraying (p<0.001), but not 24 hours after spraying (p = 0.879) as compared to before spraying, and a statistical difference was seen between 30 minutes and 24 hours (p<0.001). Specifically, between 30 minutes and 24 hours after spraying, observed contamination levels increased on 13 surfaces, most commonly on kitchen floors (4) and patient’s bed (2). A decrease in contamination levels was noted on two surfaces, including a kitchen floor and a table.

In Program B, 35 surfaces (5 households, range 5–9 surfaces/house) were positive for *E*. *coli* before spraying, compared to 23 surfaces (range 2–7) and 31 surfaces (range 5–7), 30 minutes and 24 hours after spraying, respectively (S1 Fig). Statistically, there was decreased contamination 30 minutes after spraying (p = 0.005) compared to before spraying; differences were neither significant between before spraying and 24 hours later (p = 0.018) nor between 30 minutes and 24 hours (p = 0.132). Please note p<0.017 was considered significant in post-hoc tests for multiple comparisons.

In Program C, 16 surfaces (4 households, range 3–6 surfaces/house) were positive for *E*. *coli* before spraying, compared to 10 surfaces (range 2–3) and 12 surfaces (range 2–4) 30 minutes and 24 hours after spraying, respectively (S1 Fig). Statistically, there was decreased contamination 30 minutes after spraying (p = 0.010), but not 24 hours after spraying (p = 0.515) as compared to before spraying, and a statistical difference was seen between 30 minutes and 24 hours (p = 0.008). Specifically, between 30 minutes and 24 hours after spraying, observed contamination levels increase on nine surfaces, including kitchen floors (2) and water containers (2). A decrease in contamination levels was noted on four surfaces, including floors near patient’s beds (2).

Although total coliforms were detected more often and in higher concentrations, results were similar to *E*. *coli* results (S2 Fig).

## Discussion

We conducted mixed-methods evaluations of three household spraying programs implemented in cholera response. Our results indicate that: 1) before disinfection, the highest concentrations of *V*. *cholerae* and indicator bacteria were consistently found on surfaces in food preparation areas, near the patients’ sleeping areas, and around latrine floors; 2) household spraying with chlorine can reduce bacterial contamination on household surfaces, however, effectiveness was not consistent across programs and recontamination within 24 hours was seen in the majority of households; 3) spraying programs provide opportunities for the concurrent deployment of hygiene promotion activities; and, 4) programs face challenges in locating houses as well as context-specific challenges.

The first study objective was to determine where *V*. *cholerae* were found on surfaces in cholera patient houses. The locations identified in this study as the most contaminated (food preparation areas, near the patients’ beds, and latrines) are consistent with known cholera transmission pathways [40,41] and previous evaluations of indicator bacteria and pathogens on household surfaces in England, the United States, and Tanzania [42–44]. We did not observe consistent differences in surface contamination levels prior to disinfection between private and shared latrines.

In terms of effectiveness, this study is the first we know of to provide evidence suggesting that household spraying can inactivate culturable *V*. *cholerae* and indicator bacteria on surfaces, and thus reduce cholera transmission risk. These results are in line with previous laboratory-based evaluations of chlorine spraying [26]. However, effectiveness was variable between programs. A statistically significant reduction in *V*. *cholerae* and *E*. *coli* over 24 hours was only observed in Program A. In Program B, spraying resulted in a statistically significant reduction in *V*. *cholerae* and *E*. *coli* but surfaces that were highly contaminated before spraying remained highly contaminated and reductions were not sustained over 24 hours. In Program C, no statistically significant difference was seen before/after spraying. Differences in effectiveness can be attributed to different spraying procedures. In Program A, agents were observed to systematically apply a larger volume of relatively accurately dosed 0.2 and 2.0% chlorine solution, compared to Programs B and C, where ambitious coverage objectives (20–30 houses in addition to that of the case) may have led sprayers to spend less time–and chlorine solution–at each house. Based on these differences, and a recent laboratory study where spraying chlorine was observed to achieve incomplete surface coverage [45], we hypothesize that our microbiological results reflect insufficient chlorine coverage in Programs B and C. More simply, household spraying can reduce surface contamination if chlorine solutions of appropriate concentrations are applied until surfaces are wet (i.e. contact between bacteria on surfaces and disinfectant is ensured), as reported by spraying agents and observed in Program A.

Sampling 24 hours after spraying indicated that recontamination could occur. Differences between households in observed recontamination with *V*. *cholerae* may be influenced by the presence/absence of infected individuals, which was not assessed in this evaluation. It is unclear from this evaluation whether spraying program characteristics or variable household cleaning habits are linked to recontamination with *V*. *cholerae* and indicator bacteria. Given that surfaces are typically soiled with organic matter, which inactivates chlorine [46], and recontamination is a known occurrence during water storage in houses [47], recontamination of household surfaces with indicator bacteria over time was expected.

The following (expected [29,30]) program challenges were noted: 1) spraying teams only disinfected houses of cases who reached healthcare facilities, with disinfection occurring 2–6 days following symptom onset; 2) transportation and funding were mentioned and echo international concerns about household spraying being resource-intensive; 3) locating houses was noted as difficult in Programs A and B, with agents suggesting the use of communication units (e.g. cell phones) and/or that traveling with relatives could assist in household identification; 4) context-specific challenges related to perception of the intervention noted by spraying agents, including fear of stigmatization and/or lack of trust that could lead to refusals. In comparing programs from an implementation perspective, it appeared that using spraying as a platform to deploy hygiene promotion activities at the household level was an efficient approach, as previously noted [48]. However, we were not able to assess specifically whether hygiene promotion activities responded to the needs of targeted households. Among identified program strengths, all programs used spoon-based HTH dosages that were simple and provided reasonably accurate 0.2% chlorine solutions.

Our work had several limitations, including: 1) small sample size which restricts the generalizability of observations and ability to detect associations between contamination and household variables; 2) sampling of adjacent surfaces before, 30 minutes, and 24 hours after spraying, which may have had different initial bacterial concentrations, to assess effectiveness; 3) not integrating bacterial recovery rates from surfaces into sample concentration calculations due to uncertainty, which may lead to differential underestimates of concentration levels between surface materials; and, 4) potential biases in qualitative responses and/or behaviors from household respondents (social desirability) and program staff (Hawthorne effect), neither of which impact our main results from microbiological surface sampling.

Three other study considerations are important to note: speed of deployment to carry out an evaluation in a humanitarian context, the question of *V*. *cholerae* identification and VBNC cells, and the preliminary nature of this research. Each is described further below.

In addition to results presented herein, we also deployed to Mozambique after Cyclone Idai, but arrived after spraying programs ended due to delays in securing approvals. This highlights a challenge of conducting research in some humanitarian contexts. Interviews conducted in Mozambique after the program ended support and expand upon our other results herein. Retrospective key informant interviews were conducted with three spraying program staff and five recipients of household spraying. In this program, a team of four visited patient households to carry out spraying (with different chlorine concentrations, spraying protocols, and target surfaces reported by each respondent), conduct a short survey, and test drinking water for chlorine residual. Both case and neighboring households received a hygiene kit and hygiene promotion messages, and latrines were sprayed. Spraying agents appreciated that transportation was provided. Households indicated this was their first interaction with a household spraying program, and the most commonly reported household appreciation for spraying was because it was effective against pests such as mosquitos, ants, and mice. Our experience in this and other evaluations highlights the importance of rapid research team deployment, although challenges in securing relevant approvals can delay evaluations.

The second consideration is that we intended to test surface samples for *V*. *cholerae* using viability-qPCR (with propidium monoazide pre-treatment, followed by an assay targeting the *ctxA* gene [49]) to confirm the identification of toxigenic *V*. *cholerae* O1/O139 by culture on TCBS agar and potentially detect VBNC cells. Unfortunately, viability-qPCR results were inconclusive, possibly due to inadequate preparation and/or storage of samples, undetectable concentrations, and/or detection of bacteria other than *V*. *cholerae* on TCBS agar. The use of TCBS agar for isolation of *V*. *cholerae* from clinical and environmental samples is a standard method but growth of other bacteria on that medium is possible [50,51]. In particular, sucrose-fermenting bacteria including some non-pathogenic *Vibrio* spp. and *Proteus* spp. may form yellow colonies that, despite different phenotypes, could potentially be confounded with *V*. *cholerae* if present in the tested samples [52,53]. Our evaluation results would undoubtedly have been strengthened by qPCR (or another) confirmation. We cannot exclude that our results reflect surface contamination with bacteria other than toxigenic *V*. *cholerae* O1/O139, particularly in settings such as Program C, where two patients were not considered suspected cholera cases. However, we believe that the likelihood of incorrect *V*. *cholerae* identification on TCBS agar remains low for surface samples collected in households of cholera cases in confirmed outbreak settings.

The third consideration is that this study was preliminary and not designed to answer questions of stigma, recontamination, cost-effectiveness, spraying efficacy, and epidemiological coverage. Thus, further research is needed to: 1) complete behavioral research to understand perceptions of household spraying; 2) in well-implemented programs, understand when and how recontamination occurs and if spraying should be repeated; 3) compare effectiveness, cost-effectiveness, and acceptability of household spraying and alternative interventions such as the distribution of household cleaning supplies to support decision-making; 4) complete further laboratory research with known recovery rates to assess chlorine efficacy–potentially including chlorine compounds other than HTH that might be used for spraying solutions, such as sodium dichlorosisocyanurate (NaDCC)–at inactivating *V*. *cholerae* from representative household surfaces; and, 5) refine methods to test and analyze VBNC cells, and to determine the environmental and epidemiological relevance of VBNC *V*. *cholerae* on surfaces. If future evaluations with larger sample sizes establish that household spraying is acceptable, cost-effective, and can reliably inactivate *V*. *cholerae* on household surfaces, health impact evaluations could be considered.

Overall, our results suggest that household spraying may be effective at reducing the risk of cholera transmission via fomites. While many questions remain, if responders choose to implement household spraying, our results support the following recommendations: 1) spraying agents should be trained to disinfect households systematically, including kitchen spaces, patient’s sleeping area, and latrines; 2) 0.2% chlorine should be used on household surfaces and 2.0% chlorine in latrines, soiled surfaces and, if acceptable, kitchen spaces; 3) sufficient time and resources should be available for spraying agents to meet program coverage objectives in terms of number of houses to disinfect, while performing disinfection carefully and spraying surfaces until visibly wet; 4) spraying agents should arrive at households as quickly as possible, with the assistance of communication units or patient relatives; and, 5) hygiene promotion activities should be concurrently deployed with household spraying and agents trained accordingly.

## Supporting information

S1 File Editable evaluation tools including key informant interview guides, structured observation guides, and household survey questionnaire. (XLSX)

S1 Fig (Top) *E*. *coli* concentrations on selected surfaces before, 30 minutes and 24 hours after household spraying. (Bottom) Number of surfaces where *E*. *coli* were detected (>5 CFU/100 cm^2^), by program and household.
(TIF)

S2 Fig (Top) Total coliforms concentrations on selected surfaces before, 30 minutes and 24 hours after household spraying. (Bottom) Number of surfaces where total coliforms were detected (>5 CFU/100 cm^2^), by program and household.
(TIF)

## References

[1] Sack DA, Sack RB, Nair GB, Siddique AK. Cholera. The Lancet. 2004;363(9404):223. 10.1016/s0140-6736(03)15328-7 14738797

[2] Nelson EJ, Harris JB, Glenn Morris J, Calderwood SB, Camilli A. Cholera transmission: the host, pathogen and bacteriophage dynamic. Nat Rev Microbiol. 2009 10;7(10):693–702. 10.1038/nrmicro2204 19756008 PMC3842031

[3] WHO. Ten Facts on Cholera [Internet]. 2016 [cited 2016 Oct 23]. Available from: http://www.who.int/features/factfiles/cholera/en/

[4] WHO. Cholera—Fact sheet. [Internet]. Media centre. 2016 [cited 2018 Feb 24]. Available from: http://www.who.int/mediacentre/factsheets/fs107/en/

[5] Campuzano Cuadrado P, Arcos González P. [Epidemic cholera in complex emergencies]. Rev Esp Salud Publica. 2014 4;88(2):191–201. 10.4321/S1135-57272014000200003 24914859

[6] Spiegel P B, Le P, Ververs MT, Salama P. Occurrence and overlap of natural disasters, complex emergencies and epidemics during the past decade (1995–2004). Confl Health. 2007;1:2 10.1186/1752-1505-1-2 17411460 PMC1847810

[7] Crooks AT, Hailegiorgis AB. An agent-based modeling approach applied to the spread of cholera. Environ Model Softw. 2014 12;62:164–77.

[8] UNHCR. Figures at a Glance [Internet]. UNHCR. 2018 [cited 2017 Apr 26]. Available from: http://www.unhcr.org/figures-at-a-glance.html

[9] Kelly-Hope LA. Conflict and Emerging Infectious Diseases. Emerg Infect Dis. 2008 6;14(6):1004–5. 10.3201/eid1406.080027 18507934 PMC2600301

[10] WHO. Cholera, 2018. Wkly Epidemiol Rec. 2019 Nov 29;48(94):561–80.

[11] Ali M, Nelson AR, Lopez AL, Sack DA. Updated Global Burden of Cholera in Endemic Countries. PLoS Negl Trop Dis [Internet]. 2015 6 4 [cited 2019 Aug 7];9(6). Available from: https://www.ncbi.nlm.nih.gov/pmc/articles/PMC4455997/10.1371/journal.pntd.0003832 PMC4455997 26043000

[12] GTFCC. Ending Cholera: A Global Roadmap to 2030 [Internet]. 2017. Available from: http://www.who.int/cholera/publications/global-roadmap.pdf?ua=1

[13] Codeço CT, Coelho FC. Trends in Cholera Epidemiology. PLOS Med. 2006 1 31;3(1):e42 10.1371/journal.pmed.0030042 16435891 PMC1360632

[14] Fung IC-H. Cholera transmission dynamic models for public health practitioners. Emerg Themes Epidemiol. 2014 2 12;11(1):1 10.1186/1742-7622-11-1 24520853 PMC3926264

[15] Debes AK, Ali M, Azman AS, Yunus M, Sack DA. Cholera cases cluster in time and space in Matlab, Bangladesh: implications for targeted preventive interventions. Int J Epidemiol. 2016 10 27;dyw267. 10.1093/ije/dyw267 27789673

[16] Mukandavire Z, Liao S, Wang J, Gaff H, Smith DL, Morris JG. Estimating the reproductive numbers for the 2008–2009 cholera outbreaks in Zimbabwe. Proc Natl Acad Sci. 2011 5 24;108(21):8767–72. 10.1073/pnas.1019712108 21518855 PMC3102413

[17] Mukandavire Z, Morris JG. Modeling the Epidemiology of Cholera to Prevent Disease Transmission in Developing Countries. Microbiol Spectr [Internet]. 2015 6 [cited 2018 Dec 4];3(3). Available from: https://www.ncbi.nlm.nih.gov/pmc/articles/PMC4634708/10.1128/microbiolspec.VE-0011-2014 PMC4634708 26185087

[18] Eisenberg MC, Robertson SL, Tien JH. Identifiability and estimation of multiple transmission pathways in cholera and waterborne disease. J Theor Biol. 2013 5 7;324:84–102. 10.1016/j.jtbi.2012.12.021 23333764

[19] Sugimoto JD, Koepke AA, Kenah EE, Halloran ME, Chowdhury F, Khan AI, et al Household Transmission of Vibrio cholerae in Bangladesh. Vinetz JM, editor. PLoS Negl Trop Dis. 2014 11 20;8(11):e3314 10.1371/journal.pntd.0003314 25411971 PMC4238997

[20] Tuladhar E, Hazeleger WC, Koopmans M, Zwietering MH, Beumer RR, Duizer E. Residual Viral and Bacterial Contamination of Surfaces after Cleaning and Disinfection. Appl Environ Microbiol. 2012 11 1;78(21):7769–75. 10.1128/AEM.02144-12 22941071 PMC3485719

[21] Sack DA, Sack RB, Nair GB, Siddique AK. Cholera. The Lancet. 2004;363(9404):223. 10.1016/s0140-6736(03)15328-7 14738797

[22] Merrell DS, Butler SM, Qadri F, Dolganov NA, Alam A, Cohen MB, et al Host-induced epidemic spread of the cholera bacterium. Nature. 2002 6 6;417:642 10.1038/nature00778 12050664 PMC2776822

[23] Farhana I, Hossain ZZ, Tulsiani SM, Jensen PKM, Begum A. Survival of Vibrio cholerae O1 on fomites. World J Microbiol Biotechnol. 2016 7 18;32(9):146 10.1007/s11274-016-2100-x 27430513

[24] Huq A, Rivera ING, Colwell RR. Epidemiological Significance of Viable but Nonculturable Microorganisms. In: Colwell RR, Grimes DJ, editors. Nonculturable Microorganisms in the Environment [Internet]. Boston, MA: Springer US; 2000 [cited 2018 Nov 13]. p. 301–23. Available from: 10.1007/978-1-4757-0271-2_17

[25] Oliver J. The Public Health Significance of Viable but Nonculturable Bacteria. In: Nonculturable Microorganisms in the Environment [Internet]. Colwell R.R., Grimes D.J. Boston: Springer Boston; 2000 [cited 2018 Nov 11]. p. 277–300. Available from: https://www.springer.com/us/book/9781475702736

[26] Calfee MW, Wendling M. Inactivation of vegetative bacterial threat agents on environmental surfaces. Sci Total Environ. 2013 1 15;443:387–96. 10.1016/j.scitotenv.2012.11.002 23208274

[27] Yen CH. A recent study of cholera with reference to an outbreak in Taiwan in 1962. Bull World Health Organ. 1964;30(6):811–25. 14215187 PMC2555076

[28] D’Mello-Guyett L, Gallandat K, Van den Bergh R, Taylor D, Bulit G, Legros D, et al Prevention and control of cholera with household and community water, sanitation and hygiene (WASH) interventions: A scoping review of current international guidelines. Azman AS, editor. PLOS ONE. 2020 1 8;15(1):e0226549 10.1371/journal.pone.0226549 31914164 PMC6948749

[29] UNICEF, CDC, MSF. Draft document for a position paper against chlorine spraying at households of cholera patients. 2011.

[30] UNICEF. Cholera Toolkit [Internet]. 2013 [cited 2018 Feb 24]. Available from: https://www.unicef.org/cholera_toolkit/

[31] Yates T, Vujcic JA, Joseph ML, Gallandat K, Lantagne D. Water, sanitation, and hygiene interventions in outbreak response: a synthesis of evidence. Waterlines. 2018 1 1;37(1):5–30.

[32] Neseni N, Guzha E. Evaluation of the WASH Response to the 2008–2009 Zimbabwe Cholera Epidemic and Preparedness Planning for Future Outbreaks [Internet]. 2009 [cited 2018 Feb 24]. Available from: https://www.humanitarianresponse.info/fr/operations/zimbabwe/document/evaluation-wash-response-2008-2009-zimbabwe-cholera-epidemic-and

[33] Grayel Y. Evaluation externe—Réponse d’urgence à l’épidémie de choléra en Haïti. Action Contre la Faim; 2011.

[34] Gauthier J. A real-time evaluation of ACF’s response to cholera emergency in Juba, South Suda. Action Contre la Faim; 2014.

[35] MSF. Kenya: Cholera Outbreak Spreads to Dadaab Refugee Camp [Internet]. MSF USA. 2015 [cited 2018 Feb 24]. Available from: http://www.doctorswithoutborders.org/article/kenya-cholera-outbreak-spreads-dadaab-refugee-camp

[36] Ministry of Health and Child Welfare, Zimbabwe, World Health Organization (WHO). Zimbabwe Cholera Control Guidelines. 2009.

[37] Muga R. Kenya—Cholera Control Guidelines. Disease Outbreak Management Unit; 2001.

[38] Michel E, Gaudart J, Beaulieu S, Bulit G, Piarroux M, Boncy J, et al Estimating effectiveness of case-area targeted response interventions against cholera in Haiti. Ferguson NM, Jit M, Pitzer VE, editors. eLife. 2019 12 30;8:e50243 10.7554/eLife.50243 31886768 PMC7041943

[39] Rebaudet S, Bulit G, Gaudart J, Michel E, Gazin P, Evers C, et al The case-area targeted rapid response strategy to control cholera in Haiti: a four-year implementation study. PLoS Negl Trop Dis. 2019 4 16;13(4):e0007263 10.1371/journal.pntd.0007263 30990822 PMC6485755

[40] Wagner EG, Lanoix JN. Excreta disposal for rural areas and small communities. World Health Organization, Monograph Series; 1958. 13581743

[41] Wolfe M, Kaur M, Yates T, Woodin M, Lantagne D. A Systematic Review and Meta-Analysis of the Association between Water, Sanitation, and Hygiene Exposures and Cholera in Case–Control Studies. Am J Trop Med Hyg. 2018 8 2;99(2):534–45. 10.4269/ajtmh.17-0897 29968551 PMC6090371

[42] Scott E, Bloomfield SF, Barlow CG. An Investigation of Microbial Contamination in the Home. J Hyg (Lond). 1982;89(2):279–93. 10.1017/s0022172400070819 7130703 PMC2134222

[43] Gerba CP, Tamimi AH, Maxwell S, Sifuentes LY, Hoffman DR, Koenig DW. Bacterial occurrence in kitchen hand towels. Food Prot Trends. 2014 9 1;34(5):312–7.

[44] Pickering AJ, Julian TR, Marks SJ, Mattioli MC, Boehm AB, Schwab KJ, et al Fecal Contamination and Diarrheal Pathogens on Surfaces and in Soils among Tanzanian Households with and without Improved Sanitation. Environ Sci Technol. 2012 6 5;46(11):5736–43. 10.1021/es300022c 22545817

[45] Tyan K, Jin K, Kang J, Kyle AM. Novel color additive for chlorine disinfectants corrects deficiencies in spray surface coverage and wet-contact time and checks for correct chlorine concentration. Am J Infect Control. 2018 10;46(10):1188–91. 10.1016/j.ajic.2018.03.008 29680291

[46] Rutala, Weber D. Uses of inorganic hypochlorite (bleach) in health-care facilities. Clin Microbiol Rev. 1997;10(4):597–610. 10.1128/cmr.10.4.597 PMC172936 9336664

[47] Jim Wright, Stephen Gundry, Ronan Conroy. Household drinking water in developing countries: a systematic review of microbiological contamination between source and point‐of‐use. Trop Med Int Health. 2004 1 16;9(1):106–17. 10.1046/j.1365-3156.2003.01160.x 14728614

[48] Gallandat K. Personal communications from G. Galigardini and R. Rodriguez. 2016.

[49] Blackstone GM, Nordstrom JL, Bowen MD, Meyer RF, Imbro P, DePaola A. Use of a real time PCR assay for detection of the ctxA gene of Vibrio cholerae in an environmental survey of Mobile Bay. J Microbiol Methods. 2007 2;68(2):254–9. 10.1016/j.mimet.2006.08.006 17034889

[50] Huq A, Haley BJ, Taviani E, Chen A, Hasan NA, Colwell RR. Detection, Isolation, and Identification of Vibrio cholerae from the Environment. Curr Protoc Microbiol. 2012; 10.1002/9780471729259.mc06a05s26 PMC3461827 22875567

[51] Uchiyama H. Distribution of vibrio species isolated from aquatic environments with TCBS agar. Environ Health Prev Med. 2000 1;4(4):199–204. 10.1007/BF02931258 21432485 PMC2723596

[52] Donovan TJ, van Netten P. Culture media for the isolation and enumeration of pathogenic Vibrio species in food and environmental samples. Int J Food Microbiol. 1995;26:77–91. 10.1016/0168-1605(95)00015-c 7662520

[53] Kaysner CA, DePaola A, Jones J. Bacteriological Analytical Manual—Chapter 9: Vibrio [Internet]. US Food & Drug Administration; 2004 [cited 2020 Dec 4]. Available from: https://www.fda.gov/food/laboratory-methods-food/bam-vibrio

