# Supplementary figures and images for: Household spraying in cholera outbreaks: Insights from three exploratory, mixed-methods field effectiveness evaluations
Source: PLoS Negl Trop Dis. 2020 Aug 31;14(8):e0008661. doi: 10.1371/journal.pntd.0008661 (PMC7485970; doi:10.1371/journal.pntd.0008661)

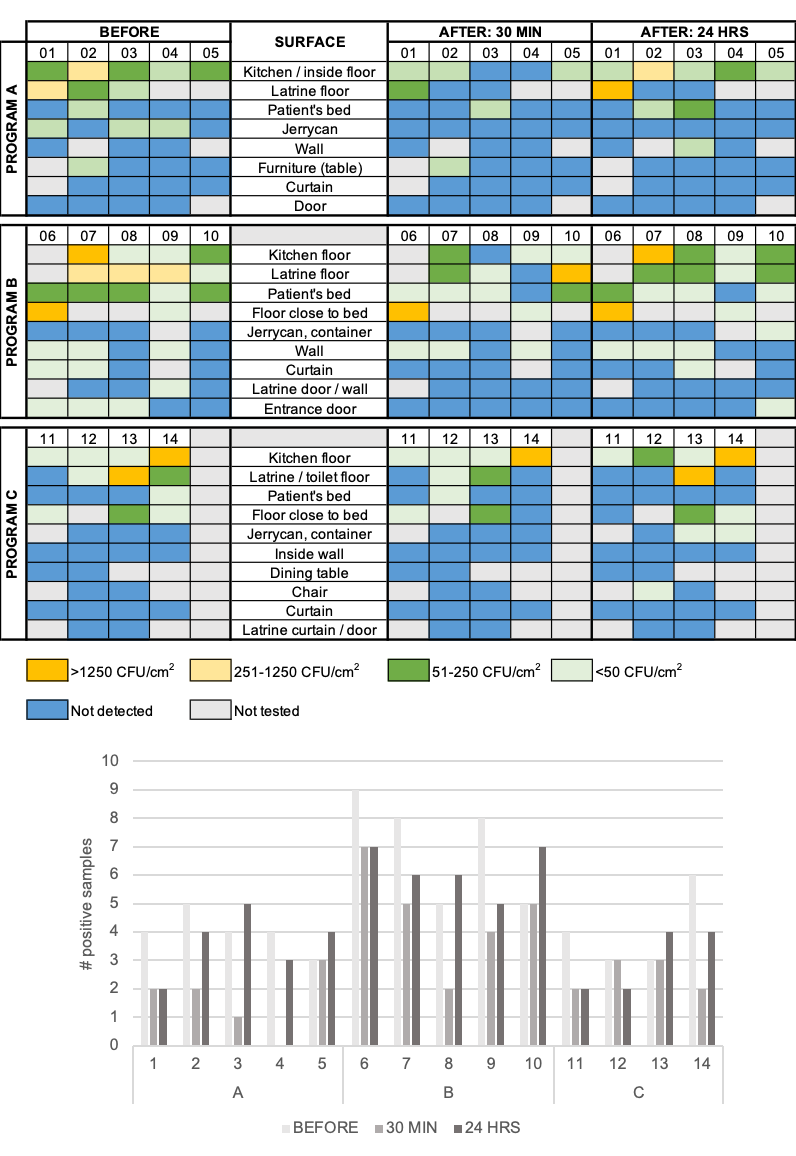

Supplement: S1 Fig — (Top) E. coli concentrations on selected surfaces before, 30 minutes and 24 hours after household spraying. (Bottom) Number of surfaces where E. coli were detected (>5 CFU/100 cm2), by program and household. (TIF) [file pntd.0008661.s002.tif]

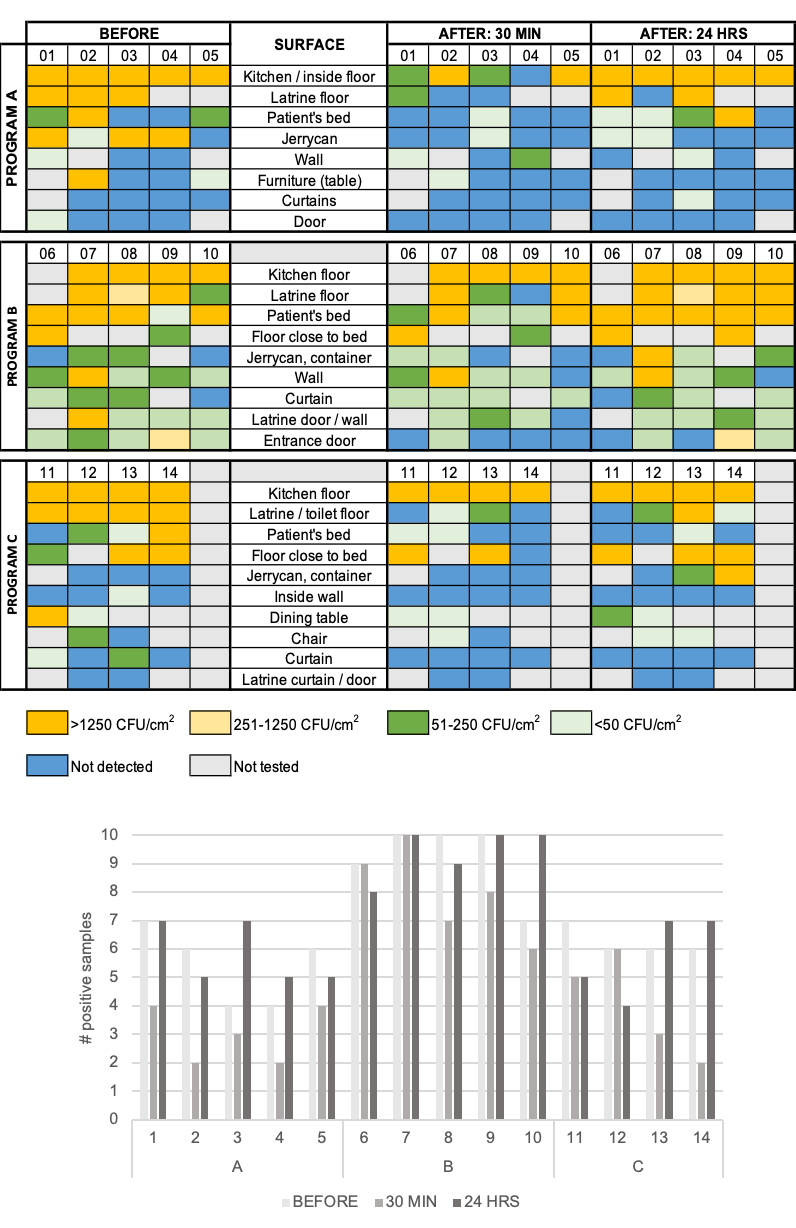

Supplement: S2 Fig — (Top) Total coliforms concentrations on selected surfaces before, 30 minutes and 24 hours after household spraying. (Bottom) Number of surfaces where total coliforms were detected (>5 CFU/100 cm2), by program and household. (TIF) [file pntd.0008661.s003.tif]
